# Vx-809, a CFTR Corrector, Acts through a General Mechanism of Protein Folding and on the Inflammatory Process

**DOI:** 10.3390/ijms24044252

**Published:** 2023-02-20

**Authors:** Michela Pecoraro, Adele Serra, Maria Pascale, Silvia Franceschelli

**Affiliations:** Department of Pharmacy, University of Salerno, Via Giovanni Paolo II, 84084 Fisciano, SA, Italy

**Keywords:** unfolded protein, endoplasmic reticular stress, oxidative stress, Vx-809 (Lumacaftor)

## Abstract

Correct protein folding is the basis of cellular well-being; thus, accumulation of misfolded proteins within the endoplasmic reticulum (ER) leads to an imbalance of homeostasis that causes stress to the ER. Various studies have shown that protein misfolding is a significant factor in the etiology of many human diseases, including cancer, diabetes, and cystic fibrosis. Misfolded protein accumulation in the ER triggers a sophisticated signal transduction pathway, the unfolded protein response (UPR), which is controlled by three proteins, resident in ER: IRE1α, PERK, and ATF6. Briefly, when ER stress is irreversible, IRE1α induces the activation of pro-inflammatory proteins; PERK phosphorylates eIF2α which induces ATF4 transcription, while ATF6 activates genes encoding ER chaperones. Reticular stress causes an alteration of the calcium homeostasis, which is released from the ER and taken up by the mitochondria, leading to an increase in the oxygen radical species production, and consequently, to oxidative stress. Accumulation of intracellular calcium, in combination with lethal ROS levels, has been associated with an increase of pro-inflammatory protein expression and the initiation of the inflammatory process. Lumacaftor (Vx-809) is a common corrector used in cystic fibrosis treatment which enhances the folding of mutated F508del-CFTR, one of the most prevalent impaired proteins underlying the disease, promoting a higher localization of the mutant protein on the cell membrane. Here, we demonstrate that this drug reduces the ER stress and, consequently, the inflammation that is caused by such events. Thus, this molecule is a promising drug to treat several pathologies that present an etiopathogenesis due to the accumulation of protein aggregates that lead to chronic reticular stress.

## 1. Introduction

Protein folding is an important physical process for forming a well-organized molecular structure, with a characteristic, thermodynamically stable conformation, by a polypeptide chain. The correct three-dimensional conformation of a polypeptide chain is indispensable for their physiological role and biological activity, whereas an incorrect spatial structure of a protein may lead to a molecule with altered physical and biochemical features [1].

Several studies have demonstrated that an incorrect protein folding is potentially toxic to cells, playing a critical role in the etiology of many human diseases, such as diabetes, atherosclerosis, cystic fibrosis, neurodegeneration, and cancer [2,3].

Further, the accumulation of proteins deployed in the ER, and its response to cellular stress, are involved in apoptosis, indicating the influence of ER on cell fate.

The ER, a network of branching tubules and flattened sacs interconnected through an enclosed space, is a skilled organelle that controls the synthesis, folding, assembly, trafficking, entering onto vesicular carriers [4], and degradation of all proteins destined to interact with organelles and extracellular space in eukaryotic cells. Furthermore, the maintenance of homeostasis by intracellular Ca^2+^ accumulation should be included among the main roles of the ER.

Alterations in ER homeostasis can determinate the accumulation of misfolded proteins, resulting in ER stress, and causing increased levels of protein synthesis; altered ubiquitination and proteasomal degradation, excess or restriction of nutrients; dysregulated calcium levels; oxidative stress; inflammatory challenges; and hypoxia [5].

To counteract ER stress, cells activate defensive mechanisms, collectively called the unfolded protein response (UPR), to alleviate the accumulation of misfolded proteins, and restore ER function.

ER stress and the activation of the unfolded protein response are associated with pathologic processes, including neurodegenerative and cardiovascular diseases as well as in cancer [6].

Despite the fact that the UPR mechanism is not yet well understood, it is commonly accepted that the UPR is regulated by three major sensors: double-stranded, RNA-activated protein kinase-like ER kinase (PERK), activating transcription factor 6 (ATF6), and inositol-requiring enzyme 1 (IRE1) [7].

Under physiological conditions, all these three sensors remain in their inactive conformation by binding to the molecular chaperone, Grp78/BiP, that actively promotes protein folding, the import of polypeptides, and the export of misfolded proteins towards the ER-associated protein degradation (ERAD). When misfolded proteins accumulate within the ER lumen, Grp78/BiP dissociates from PERK, IRE1, and ATF6, causing their activation. IRE1 and PERK switch from a monomeric inactive conformation to active oligomers [8].

Indeed, PERK undergoes dimerization and autophosphorylation, and subsequently phosphorylates the eukaryotic initiation factor 2α (eIF-2α), to prevent the initiation of translation and prevent the syntheses of new proteins in the cytoplasm. PERK-phosphorylated eIF2α also induces the activation of transcription factor 4 (ATF4), which is a key product of UPR-mediated gene expression. ATF4 translocates to the nucleus, triggering the transcription of genes required to restore ER homeostasis, inducing the cell survival. Moreover, PERK hyperactivation promotes cell apoptosis by increasing ATF4 and the C/EBP homologous protein (CHOP) [7,9]. Additional studies also suggest the role of CHOP in promoting protein synthesis to cause oxidative stress, leading to apoptosis [10].

In response to ER stress, BiP separates from ATF6, a transcription factor of the cAMP response element-binding protein (CREB)/ATF family, which moves to the Golgi apparatus, where it is cleaved into its active form and translocates to the nucleus where it induces the transcription of ER stress-response genes [7,11].

**Figure 1:**
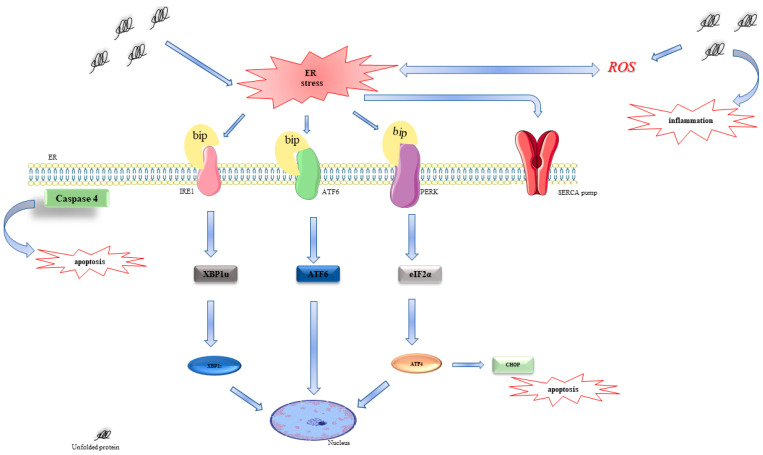
Overview of the unfolded protein response pathway in response to ER stress. Upon the accumulation of misfolded proteins in the ER, BiP is released from the ER membrane to induce PERK dimerization and its subsequent auto-phosphorylation. In turn, activated PERK phosphorylates eIF2α, leading to global translation attenuation. ATF4 provides the transcriptional signal to restore ER homeostasis; however, it can also induce proapoptotic CHOP. Moreover, IRE1α undergoes oligomerization and autophosphorylation and splices XBP1 mRNA to release transcriptionally active XBP1. XBP1s activate a transcriptional program to restore ER homeostasis. Finally, BiP dissociation enables translocation of ATF6 to the Golgi, where cleavage of this protein results in the release of transcriptionally active ATF6f to restore ER homeostasis and support ERAD.

IRE1 has a serine/threonine kinase activity and a ribo-nuclease (RNase) region on the cytoplasmic side (C-terminus). When ER stress is sensed, IRE1 is activated by autophosphorylation, leading to the removal of a 26-nucleotide intron from the premature unspliced XBP1 (XBP1-u) mRNA form to produce the spliced XBP1 (XBP1-s) form. XBP1-s is a transcription factor that moves to the nucleus, binding to the specific promoter elements, such as the ER stress response element (ERSE) and UPR elements, to trigger transactivation of UPR target genes, such as those whose products are involved in protein folding, secretion, and degradation [12,13]. Increasing evidence suggests that IRE1α regulates a variety of cell physiologies, including metabolism, immunity, cell differentiation, and apoptosis [14] (Figure 1).

However, if ER stress is prolonged to the extent that UPR is unable to cope with unfolded proteins, UPR activates apoptotic downstream pathways through ATF6, PERK, and IRE1 signaling pathways [8].

ER stress and oxidative stress are event closely linked to cell homeostasis and apoptosis; in a stressed ER, dysregulated disulfide bond formation and breakage occur that result in reactive oxygen species (ROS) accumulation, which in turn causes oxidative stress. Meanwhile, ER stress determines mitochondrial dysfunction and increases mitochondrial ROS production [15].

ROS, produced from the ER or other sources, can target the ER calcium channels, leading to ER Ca^2+^ release. Calcium^+^ released from the ER is taken up by the mitochondria, then stimulates mitochondrial metabolism and ROS production [16].

The ROS production, the activation of the transcription factor nuclear factor-kB (NF-kB), and the induction of acute-phase proteins have been linked to both ER stress and inflammatory responses. Indeed, the ER stress response and inflammation are interconnected through various additional mechanisms [17].

**Figure 2:**
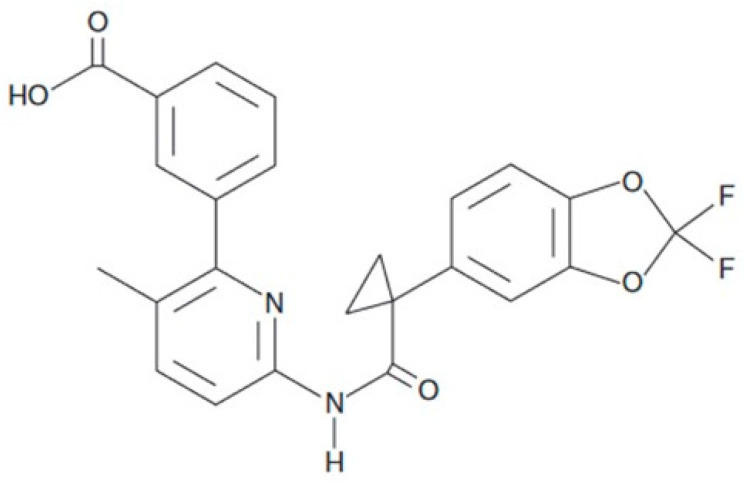
Vx-809 structure.

Lumacaftor (Vx-809) (Figure 2) is the first drug approved for the treatment of cystic fibrosis in patients homozygous for the most common cystic fibrosis transmembrane Conductance Regulator (CFTR), mutated F508del-CFTR. This modification enhances the processing of F508del-CFTR and its transport to the cell surface. Usually, the defective protein is degraded before reaching the cell membrane, where it must be used to carry out the transepithelial transport of chlorine. Consequently, this severe mutation is associated with an impaired CFTR function.

Vx-809 was selected by high-throughput screening and it is based on a di-fluorobenzodioxolyl-cyclopropane linked to a substituted arylpyridine through an amide linkage (Figure 1). Its mode of action is yet to be elucidated but it may stabilize the folding of the molecule either by direct binding to NBD1 or through promoting interactions between TMD1 and NBD1. Interestingly, Vx-809 was shown to rectify trafficking of another impaired ABC transporter (ABCA4), which shares large homology with CFTR NBDs, indicating that Vx-809 is not CFTR-specific [18,19,20].

Mutated CFTR is recognized as a misfolded protein trapped in the ER, causing stress. The excessive or long-termed ER stress results in apoptotic cell death, involving nuclear fragmentation and chromatin condensation [21]. However, the potential beneficial effect of Vx-809 could expand further. The drug’s ability to be a corrector of the mutated CFTR channel protein could also be applied to other proteins not correctly folded, demonstrating that it can also be used in other pathologies.

Based on these hypotheses, the aim of this work is aimed to postulate new potential applications of the Vx-809, not only in restoring the mutated CFTR protein functionality, but also in other disorders that manifest their mechanism of pathogenesis through protein misfolding.

## 2. Results

### 2.1. Vx-809 Interferes with UPR Activation

Under ER stress conditions, misfolded proteins accumulate in the ER lumen, causing a pathologic process which stimulates the UPR pathway through the ER chaperone’s activation, like Grp78/BiP. Thus, since Grp78/Bi presides mainly in the ER, it plays a pivotal role in the cellular stress of various diseases [22].

To test the hypothesis of the involvement of Vx-809 in misfolding protein, we analyzed, through Western blotting analyses on cell lysates, the activation state of UPR signaling components, such as Grp78/BiP, ATF4 after inducing ER stress in adenocarcinomic human alveolar basal epithelial cells (A549), and malignant melanoma (A375).

In our experiments, we used Thapsigargin (TG), an inhibitor of Ca^2+^-ATPase pump, previously characterized as an agent that induces ER stress [23].

Western blotting analysis on A549 and A375 extracts revealed that TG treatment significantly (*p* < 0.005) increased Grp78/BiP levels, confirming the onset of a reticular stress condition.

**Figure 3:**
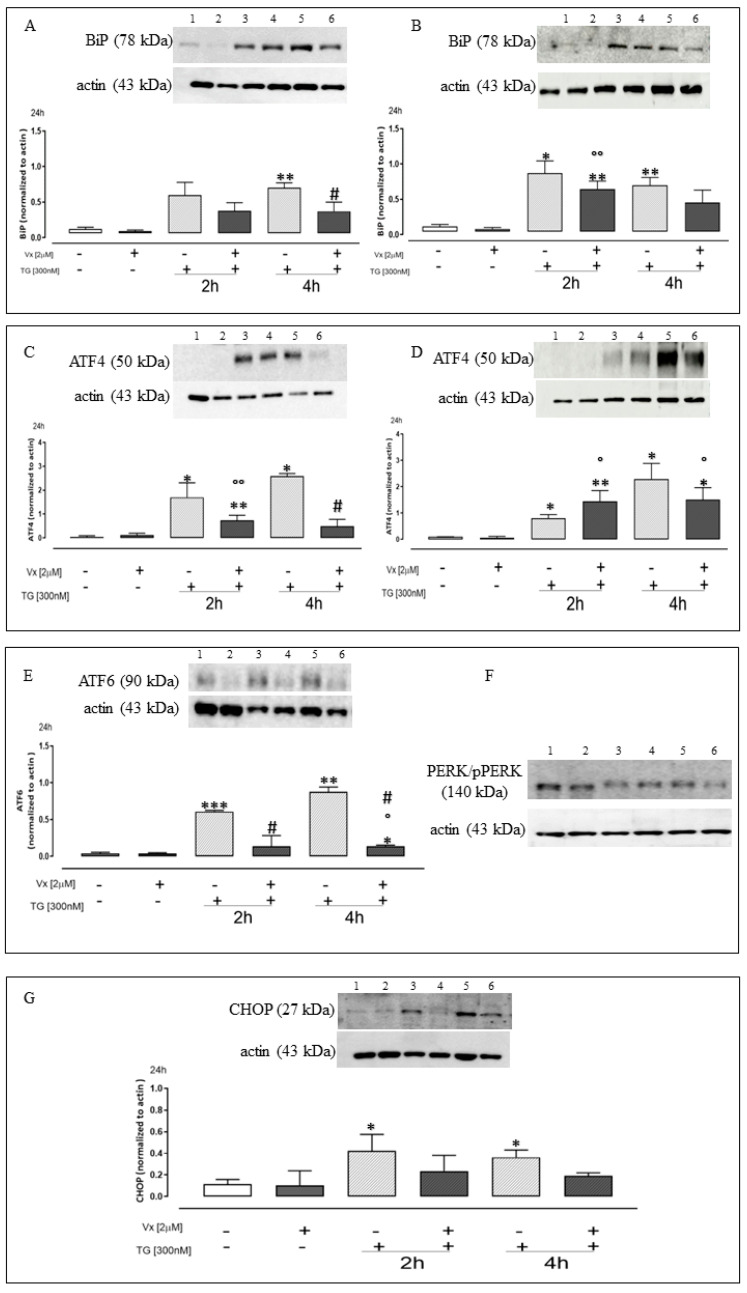
Vx-809 interferes with UPR activation. Western blot analysis: 1 = ctrl, 2 = Vx-809, 3 = TG for 2 h, 4 = Vx-809 after pre-treatment with TG for 2 h, 5 = TG for 4 h, 6 = Vx-809 after pre-treatment with TG for 4 h. Cells were pretreated with 300 nM TG for 2 or 4 h to induce ER stress. Subsequently, 2 µM of the corrector Vx-809 were administered for 24 h. BiP and ATF4 expressions on A549 (**A**,**C**) and on A375 (**B**,**D**) were detected by Western blot analysis as well as in ATF6 (**E**), PERK/pPERK (**F**), and CHOP (**G**) on A549 cells. Actin protein expression was used as a loading control. Results are expressed as mean ± S.E.M. from at least three independent experiments each performed in triplicate. Data were analyzed by a Mann–Whitney U test. * *p* < 0.05, ** *p* < 0.005, and *** *p* < 0.001 vs. non-treated cells; ° *p* < 0.05 and °° *p* < 0.005 vs. Vx-809–treated cells; # *p* < 0.05 vs. TG-treated cells.

Vx-809 administration reduced TG-induced Grp78/BiP over-expression, significantly (*p* < 0.05) at 4 h pretreatment, as shown in Figure 3A,B, respectively.

ATF4 is a transcription factor that regulates a wide range of genes and plays a crucial role in cell adaptation to stress conditions [24]. Vx-809 treatment significantly (*p* < 0.005) reduced TG-induced ATF4 levels, at both pretreatment times, confirming its effect on ATF4 activation in A549 (Figure 3C) and in A365 cells (Figure 3D).

Several studies show that the deficiency of the ATF6 protein results in cells’ inability to manage ER stress response [25,26,27]. Data on A549 cells showed that ATF6 expression significantly increased (*p* < 0.001) in cells pretreated at 2 h and 4 h with TG compared to control cells, confirming the reticular stress presence in our experimental model. Vx-809 treatment significantly (*p* < 0.05) reduced TG-induced ATF6 levels, at both TG-pretreatment times (Figure 3E). Further, the presence of an insult to the ER homeostasis leads to the detachment of the Grp78/BiP chaperon from the PERK protein, which promotes its phosphorylation.

To evaluate PERK activation, we analyzed the phosphorylated PERK form (p-PERK) by Western blotting on a A549 line cell. In particular, PERK phosphorylation was recognized by immunoblots, as depicted in Figure 3F, showing the band-shift of p-PERK in Western blot analyses as a consequence of the higher molecular weight acquired by the auto-phosphorylation [28,29]. As expected, in untreated and treated Vx-809 cells, p-PERK form was not detected, but only after treatment with the ER stress inducer, TG. Further, Vx-809 administration caused a reduction in the band-shift of p-PERK, at 4 h TG pretreatment.

Finally, we evaluated the CHOP expression, widely involved in the ER stress processes. As shown in Figure 3G, Western blot analysis performed on A549 cell lysates showed that TG-treatment induces a significant (*p* < 0.05) increase of CHOP levels compared to control cells, corroborating the presence of reticular stress. Vx-809 treatment reduced the expression of the protein in our experimental model, thus helping the cell to restore reticular homeostasis.

### 2.2. Vx-809 Counteract Thapsigargin-Induced Apoptotic Response

**Figure 4:**
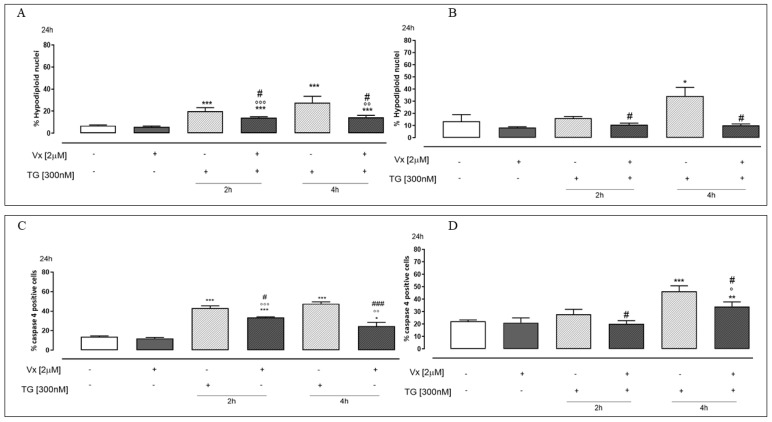
Vx-809 interferes in cell death. Cells were pretreated with 300 nM TG for 2 or 4 h to induce ER stress. Subsequently, 2 µM of the corrector Vx-809 were administered for 24 h. Then, A549 (**A**) and A375 (**B**) were stained by propidium iodide and the fluorescence of individual nuclei was measured by flow cytometry. Results are expressed as mean ± S.E.M. of the percentage of hypodiploid nuclei from at least three independent experiments each performed in duplicate. Panels (**C**,**D**) show caspase 4 content detected by flow cytometry analysis, respectively, in A549 and A375 cells. Data were expressed as mean ± S.E.M. of the percentage of caspase 4 positive cells from at least three independent experiments each performed in duplicate. Data were analyzed by a Mann–Whitney U test. * *p* < 0.05, ** *p* < 0.05, and *** *p* < 0.001 vs. non-treated cells; ° *p* < 0.05, °° *p* < 0.005, and °°° *p* < 0.001 vs. Vx-809–treated cells; # *p* < 0.05 and ### *p* < 0.001 vs. TG-treated cells.

The main role of the UPR system is to re-establish reticular homeostasis when altered. However, when restoration of the physiological conditions of the ER is not possible, the best way forward is apoptosis [25]. Moreover, since CHOP expression has been reported as a molecule involved in ER stress-induced apoptosis [30], we evaluated, in our experimental model, the percentage of hypodiploid nuclei and caspase 4 activation. Figure 4 shows that Vx-809 causes a significant (*p* < 0.05) reduction of the percentage of hypodiploid nuclei in TG-pretreated A549 (Figure 4A) and A375 (Figure 4B) cells.

Caspase 4 is localized in the ER membrane and is cleaved in ER stress conditions [21]. Flow cytometry analysis showed a significant (*p* < 0.001) reduction of caspase 4 levels in Vx-809–treated A549 (Figure 4C) and A375 cells (Figure 4D), in ER stress conditions, implying that Vx-809 restores reticular homeostasis.

### 2.3. Vx-809 Is Involved in Intracellular and Mitochondrial ROS Production

**Figure 5:**
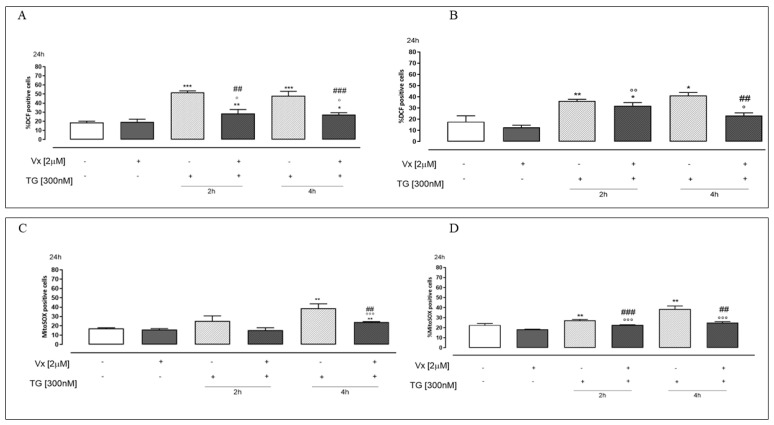
Vx-809 interferes in the ROS production. Cells were pretreated with 300 nM TG for 2 or 4 h to induce ER stress. Subsequently, 2 µM of the corrector Vx-809 were administered for 24 h. ROS generation was evaluated by the probe 2′,7′dichlorofluorescein diacetate (H_2_DCF-DA) in A549 (**A**) and in A375 (**B**) cells. ROS production was expressed as mean ± SEM of the percentage of DCF-positive cells of at least three independent experiments each performed in duplicate. Superoxide production by mitochondria was evaluated by means of the probe MitoSOX Red in A549 (**C**) and A375 (**D**) cells by flow cytometry analysis. Mitochondrial superoxide production was expressed as mean ± S.E.M. of the percentage of MitoSOX-positive cells of at least three independent experiments each performed in duplicate. Data were analyzed by a Mann–Whitney U test. * *p* < 0.05, ** *p* < 0.005, and *** *p* < 0.001 vs. untreated cells; ° *p* < 0.05, °° *p* < 0.005, and °°° *p* < 0.001 vs. Vx-809–treated cells; ## *p* < 0.005 and ### *p* < 0.001 vs. TG-treated cells.

During the accumulation of misfolded proteins in the ER lumen, the UPR response promotes the production of ROS in the ER, which has been shown in many physiological and pathological conditions. Cytofluorimetric analysis by fluorescent probe DCHF-DA showed that Vx-809 administration significantly (*p* < 0.001) decreased cytosolic ROS production in A549 (Figure 5A) and A375 (Figure 5B) TG-treated cells. Since the induction of ER stress from the accumulation of unfolded proteins reveals a much broader functional link between chaperones and mitochondrial metabolism, and mitochondria represent an important source of ROS, we evaluated the mitochondrial ROS generation [31]. Results obtained using MitoSOX red showed that mitochondrial ROS production was significantly (*p* < 0.001) lower in Vx-809–treated A549 (Figure 5C) and A375 (Figure 5D) cells after pretreatment with TG at 4 h.

### 2.4. The “Corrector” Vx-809 Reduces Thapsigargin-Induced Ca^2+^ Homeostasis Dysregulation

Most ER-associated proteins participate in maintaining Ca^2+^ homeostasis. For example, molecular chaperones such as BiP or other folding enzymes contribute to Ca^2+^ buffering in the ER lume. Moreover, disruption of Ca^2+^ homeostasis in the ER leads to UPR activation. Minor disturbances of ER Ca^2+^ homeostasis have been linked to many human diseases such as cardiac diseases and in many neuropathies [32].

**Figure 6:**
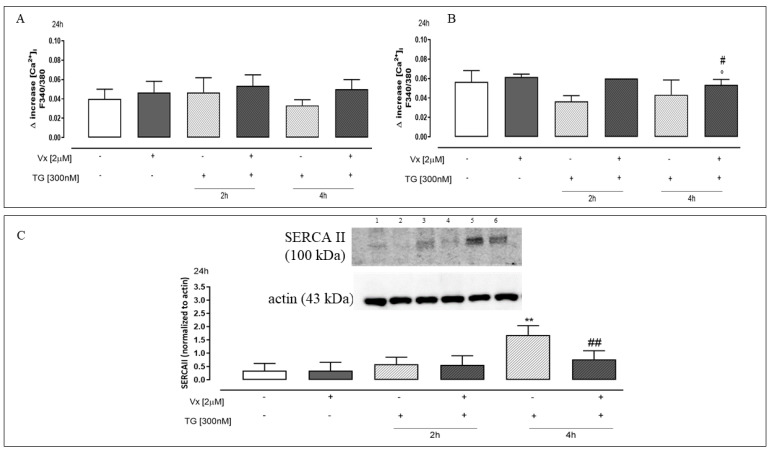
Vx-809 interferes with calcium concentrations. Western blot analysis: 1 = ctrl, 2 = Vx-809, 3 = TG for 2 h, 4 = Vx-809 after pre-treatment with TG for 2 h, 5 = TG for 4 h, 6 = Vx-809 after pre-treatment with TG for 4 h. Cells, to induce ER stress, were pretreated with 300 nM TG for 2 or 4 h. Subsequently, 2 µM of the corrector Vx-809 were administered for 24 h. The effect of Vx-809 after ER stress conditions on the reticular calcium pool was evaluated on cells in a calcium-free medium in the presence of Thapsigarging (1 nM) (Panel (**A**)), while intracellular calcium content was evaluated by means of Ionomycin (1 μM) (Panel (**B**)). Results are expressed as mean ± S.E.M. of the delta (δ) increase of FURA 2 ratio fluorescence (340/380 nm) from at least three independent experiments each performed in duplicate. SERCAII expression was detected by Western blot analysis; actin protein expression was used as a loading control. Results are expressed as mean ± S.E.M from at least three independent experiments each performed in duplicate (Panel (**C**)). Data were analyzed by a Mann–Whitney U test. ** *p* < 0.005 vs. untreated cells; ° *p* < 0.05 vs. Vx-809–treated cells; # *p* < 0.05 and ## *p* < 0.005 vs. TG-treated cells.

To evaluate the Vx-809 treatment effects on intracellular Ca^2+^ levels in our experimental model, A549 cells, treated as previously described, were loaded with the fluorescent dye FURA2-AM in Ca^2+^-free incubation medium (containing 0.5 mM EGTA). As depicted in Figure 6A, the delta increase in [Ca^2+^]_i_ obtained by Thapsigargin (1 nM), in Vx-809–treated cells previously pretreated with TG at 4 h, was higher than in 300 nM TG- treated cells, highlighting the restoration of calcium accumulation in the ER. Thus, we hypothesize that the Vx-809 corrector improves calcium homeostasis, following reticular stress. Similarly, our results indicate that Vx-809 treatment, after reticular stress, causes a significant (*p* < 0.05) growth of the delta increase in [Ca^2+^]_i_ induced by Ionomycin (1 μM), versus TG-treated cells (Figure 6B) in our experimental model, assuming the beneficial effect of the corrector on calcium homeostasis.

The SERCAII pump (Sarco/Endoplasmic Reticulum Ca^2+^-ATPases) sequesters Ca^2+^ ions within the ER, which are then released into the cytosol in response to a variety of physiological events. Thus, SERCAII is a key regulator of intracellular Ca^2+^ homeostasis, among all the regulatory systems involved [33]. 

We therefore analyzed the expression of SERCAII levels in A549 cell lines. Data obtained by Western blot analysis showed a significant (*p* < 0.005) reduction of SERCAII expression in Vx-809-treated cells, previously pretreated with TG at 4 h (Figure 6C). Our hypothesis is that the SERCAII pump, moving calcium into the ER, increases its expression following the reticular stress induction, as a defense mechanism, since an excess of calcium present inside the cytosol is harmful to the cell itself.

### 2.5. Effect of Vx-809 on the Inflammatory Pathway

**Figure 7:**
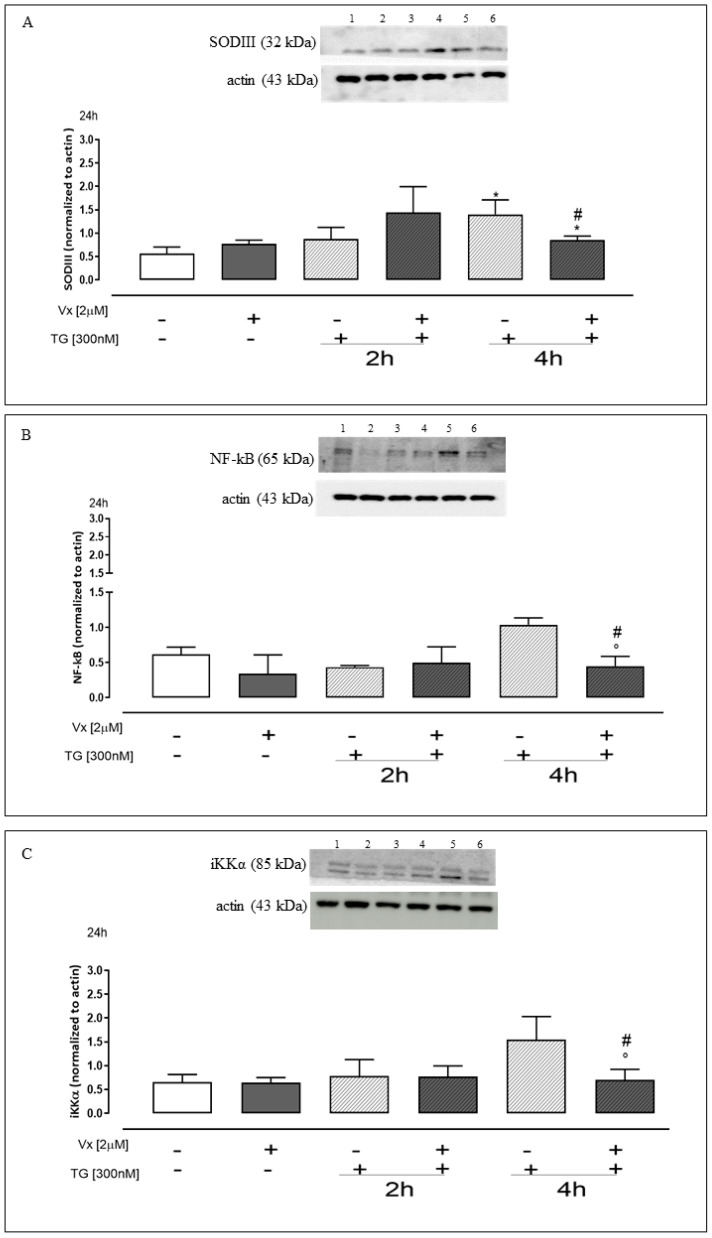
The effect of Vx-809 on the inflammatory pathway. Western blot analysis: 1 = ctrl, 2 = Vx-809, 3 = TG for 2 h, 4 = Vx-809 after pre-treatment with TG for 2 h, 5 = TG for 4 h, 6 = Vx-809 after pre-treatment with TG for 4 h. Cells were pretreated with 300 nM TG for 2 or 4 h to induce ER stress. Subsequently, 2 µM of the corrector Vx-809 were administered for 24 h. SODIII (**A**), NF-kB (**B**), and iKKα (**C**) expressions were detected by Western blot analysis. Actin protein expression was used as a loading control. Results are expressed as mean ± S.E.M. from at least three independent experiments each performed in triplicate. Data were analyzed by a Mann–Whitney U test. * *p* < 0.05 vs. non-treated cells; ° *p* < 0.05 vs. Vx-809–treated cells; # *p* < 0.05 vs. TG-treated cells.

In oxidative stress conditions, cells increase the expression of antioxidant enzymes such as superoxide dismutase (SOD), which neutralizes free radicals [21]. Therefore, we analyzed SOD III, which is amply expressed in the lungs and its concentration is increased by pro-inflammatory cytokines. Western Blotting analysis showed a significant (*p* < 0.05) reduction of SOD III expression in Vx-809–treated cells after reticular stress induction versus TG-treated cells, like depicted in Figure 7A.

Recently, increased ER stress and ROS production have been observed in several diseases, suggesting that ROS has a crucial regulator of ER stress, although the molecular link between ROS and components of the ER stress machinery are still obscure. The excessive release of ROS may result in the development of inflammation [34].

Several studies showed that the inflammatory pathway is regulated by the nuclear factor-kB (NF-kB)/iKKα, whose activation can be caused by increased ROS levels [21,35]. Thus, we chose to analyze the expression NF-kB and iKKα levels in A549 cell lines treated as above. In TG-A549-treated cells at 4 h, we observed high levels of both proteins, confirming the establishment of a stress condition in our experimental model. After stress induction and Vx-809 administration for 24 h, cells underwent a significant (*p* < 0.05) reduction in NF-kB (Figure 7B), hypothesizing a non-translocation in the nucleus, and iKKα (Figure 7C) expression, compared to TG treatment alone.

## 3. Discussion

ER is a pivotal organelle involved in biosynthesis of proteins, post-translational modification, folding, and assembly of newly synthesized proteins and cellular homeostasis. Unfolded protein accumulation leads to ER stress, followed by an adaptive response via the activation of the UPR, PERK, IRE1α, and ATF6 pathways [36].

It is well known that the ER stress response regulates the onset, progression, and severity in a variety of pathologies such as cancer, diabetes, atherosclerosis, obesity, and neurodegenerative diseases [37].

Lumacaftor (Vx-809) is a small molecule used to treat cystic fibrosis disease functioning as a corrector of mutated CFTR protein, acting on the folding and trafficking of CFTR proteins and increasing their amount on the cell surface [21,38].

In the present study, we show for the first time that Vx-809 is involved in the mechanism of correction of misfolded proteins. We demonstrated that Vx-809 not only restores the misfolding responsible for cystic fibrosis, but actively participates in the correction of stress-induced misfolding of the ER.

ER stress was induced by Thapsigargin, a well-known stress inducer [39], that causes a depletion of calcium from the ER lumen followed by accumulation of unfolded or misfolded proteins in the ER.

In our experimental model, Vx-809 counteracts the activation of the UPR mechanism, most likely because it restores protein folding. In fact, as demonstrated by Western blot analysis, Vx-809 significantly reduced the first ER chaperone protein involved, Grp78/BiP, under ER stress condition.

Residing primarily in the ER, Grp78/BiP plays critical roles in the cellular stress of various diseases. In addition to facilitating proper folding of proteins, preventing intermediates from aggregating, and targeting misfolded proteins for proteasome degradation, Grp78/BiP also binds Ca^2+^ and serves as a signaling regulator of ER stress [22].

In our experimental model, the results indicate that the Vx-809 corrector is involved in UPR pathway, significantly reducing the expression of ATF4, ATF6, pPERK, and CHOP, as showed by Western blot analyses. Taken together, these results demonstrate that Vx-809 corrector restores a reticular physiological condition caused by a stressful situation.

Under ER stress conditions, the accumulation of unfolded proteins causes an enhanced expression of CHOP that promotes apoptotic cell death [25]. Moreover, it has been reported that in cultured human cells, the activation of caspase 4 is precipitated and reduced by ER stress [21]. In this study, we demonstrated that Vx-809 administration likely acts on protein folding, reducing the apoptotic process, as shown by FACs analysis. Furthermore, in our experimental model, countering the ER stress, cytofluorimetric analysis showed a reduction of caspase 4 levels.

ER stress and oxidative stress interact in a variety of physiological and pathological scenarios. Moreover, the key defense networks that assist cells to adapt and survive stress conditions induced by biochemical, physiological, and pathological stimuli are triggered by ER stress, oxidative stress, and inflammatory responses. Chronic ER stress, oxidative stress, and inflammation, on the other hand, have been linked to the onset and progression of a wide range of human disorders [15]. Here, we have shown that treatment with Vx-809, in a reticular stress condition, causes a reduction in both intracellular and mitochondrial ROS levels, suggesting that it can reduce the oxidative stress condition.

Incorrect protein folding, hence, causes ER stress and increased ROS production, resulting in the release of calcium from the ER. Furthermore, elevated ROS levels can induce cell death by association with calcium overload in the cell [40]. Our data show in our experimental model that the proper folding of unfolded proteins, obtained by administering Vx-809, causes a restoration of the regulation of calcium homeostasis due to the recovery of reticular and cytosolic Ca^2+^ accumulation and reduces the levels of free Ca^2+^ in A549 cells. Furthermore, the improvement of reticular Ca^2+^ storage is also regulated by the SERCA II pump, as shown by Western blot analysis, suggesting that Vx-809 causes a reduction of SERCA II expression, assuming that its expression is high under reticular stress as a defense mechanism to reduce the cytoplasmic levels of calcium to be stored inside the ER. Treatment with the corrector seems to reduce stressful reticular conditions and, therefore, cells no longer need to sequester calcium from the cytosol, reducing the expression of the calcium pump.

Under ER stress conditions, when ROS levels are significantly high, cells protect themselves by increasing the expression of antioxidant proteins, such as SODIII, to cope with the damage caused by radical species.

Our results in our experimental model confirm the increase of SODIII expression, when cells were treated with Thapsigargin at 4 h, while a significant reduction occurred in the cells when Vx-809 was administered. Thus, taken together, our results strongly suggest that the Vx-809 corrector reduces the expression of antioxidant proteins by restoring cellular homeostasis.

In addition, Vx-809 seems to play a relevant role in the inflammatory process associated with stressful ER condition, caused by protein misfolding. More, our results show that in our experimental model Vx-809 treatment induces a significant reduction of IKKα with a concurrent reduction of NF-kB expression. Thus, the corrector inhibits the NF-kB pathway, preventing the transcription of pro-inflammatory cytokines.

Altogether, these results are very promising indeed: treatment with Vx-809 could, in fact, be considerably extended to other specific pathologies. The beneficial effects of this corrector in the cystic fibrosis disease are widely reported in the literature; thus, the experimental evidence we have reported in this work is the starting point for a potential application of Vx-809 in other pathologies as well, such as Parkinson’s and Alzheimer’s diseases, which have their origin in protein misfolding.

## 4. Materials and Methods

### 4.1. Reagents

Vx-809 (S1565) was purchased from Selleckchem (Houston, TX, USA). Monoclonal antibodies used were the anti-ATF4 (10835-1-AP, proteintech), anti-ATF6 (658805, cell signaling), anti-CHOP (TA802218, origene), anti-PERK (3192, cell signaling), anti-Grp78/BiP (3177, cell signaling), anti-MnSODIII (sc-271170), anti-actin (TA811000, origene), anti-iKKα (sc-7606), anti-caspase 4 (sc-1229), anti-NF-kB (MA5-15181), and anti-SERCAII (sc-376235). Secondary antibodies (anti-rabbit, A120-101P and anti-mouse, A90-137P) were purchased from Bethyl Laboratories (Montgomery, TX, USA). Texas red-conjugated secondary antibody (T6390 and PA1-28662) was bought by Thermo Fisher Scientific (Waltham, MA, USA). MitoSOX Red Mitochondrial Superoxide Indicator was bought from Invitrogen, ThermoFisher. Thapsigargin, H_2_DCF-DA, FURA 2AM, Ionomicin, and propidium iodide were purchased from Sigma-Aldrich (St. Louis, MO, USA).

### 4.2. Cell Culture

Adenocarcinomic human alveolar basal epithelial cells (A549) and malignant melanoma (A375) were subcultured weekly in 75 cm^2^ sterile culture flasks with Dulbecco’s modified Eagle’s Medium (DMEM; Euroclone, Pero (MI), Italy) containing 10% fetal bovine serum (FBS; Euroclone), 100 U/mL penicillin, and 100 μg/mL streptomycin, and glutamine (2 mM) in a humidified atmosphere of 5% CO_2_ at 37 °C. Cells were always used at less than 80% of confluence.

### 4.3. Experimental Protocol

Both cell lines were seeded and, after adhesion (24 h), were pretreated with 300 nM TG for 2 or 4 h to induce ER stress. Subsequently, after removing the TG, fresh medium was added to the cells with and without Vx. The corrector Vx-809 (2 µM) was administered for 24 h and then cell lysates were collected after 24 h of treatment for both Vx- and TG-treated cells. Experiments were performed on the A549 cells, while only a few experiments were performed on melanoma cells, used to confirm our hypothesis of the Vx-809 involvement also on another cell line.

### 4.4. Protein Extraction and Western Blot Analysis

Cells (7 × 10^5^ cells/plate) were seeded in 100 mm tissue culture plates and were treated, after 24 h, as described in experimental protocol.

Total proteins were extracted from cells by freeze/thawing in RIPA buffer (containing 1 mm EDTA, 150 mM NaCl, 20 mM K^+^-Hepes pH 7.5, 1% IGEPAL, and protease inhibitor cocktail). Then, cells were centrifuged at 14,000 rpm for 15 min at 4 °C. Protein concentration was analyzed by a Bradford assay and 30 µg of protein were run on 8–10% acrylamide gel and separated by SDS-PAGE, under denaturing conditions, and transferred to nitrocellulose membranes using a minigel apparatus (Bio-Rad Laboratories, Richmond, BC, Canada). Blots were then blocked in Tris-buffered saline, containing 5% nonfat dry milk for 1 h at room temperature, and incubated overnight with specific primary antibodies at 4 °C. Actin was used as a loading control. After washes in PBS/0.1% Tween, the right secondary antibody was added for 1 h at room temperature. The immunoreactive protein bands were then visualized using enhanced chemiluminescence reagents (ECL) and blot imaging (LAS 4000; GE Healthcare, Milano (MI), Italy). Western blot data were quantified using ImageJ Software [21,41].

### 4.5. Measurement of Intracellular Calcium Signaling

Intracellular calcium concentrations were analyzed using the fluorescent indicator dye Fura 2-AM, the membrane-permeant acetoxymethyl ester form of Fura 2. Briefly, cells (3 × 10^4^ cells/plate) were planted in 6-well tissue culture plates and treated as described above. After treatment period, cells were washed in phosphate buffered saline (PBS) and resuspended in 1 mL of Hank’s balanced salt solution (HBSS) containing 5 μM Fura 2-AM for 45 min [42]. Afterwards, cells were washed with the same buffer to remove excess Fura 2-AM and incubated in calcium-free HBSS/0.5 mM EGTA buffer for 15 min to push Fura 2-AM hydrolysis into its active-dye form, Fura 2. Then, A549 cells were transferred to the spectrofluorometer (Perkin-Elmer LS-55, Milano (MI), Italy). Treatment with Thapsigargin (1 nM) or with Ionomycin (1 μM) was carried out by adding the right concentrations of each substance into the cuvette in calcium-free HBSS/0.5 mM EGTA buffer. The excitation wavelength was alternated between 340 and 380 nm, and emission fluorescence was recorded at 515 nm. The ratio of fluorescence intensity of 340/380 nm (F340/F380) was strictly related to intracellular free calcium, as previously reported [43]. Results were expressed as a delta (Δ) increase in the fluorescence ratio (F340/F380 nm) induced by Thapsigargin—basal fluorescence ratio (F340/F380 nm) or Ionomycin—basal fluorescence ratio (F340/F380 nm).

### 4.6. Intracellular and Mitochondrial ROS Release Measurement

Intracellular ROS production was measured by the probe 2′,7′-dichlorofluorescein-diacetate (H_2_DCF-DA). The assay is based on the cellular esterase action on H_2_DCF-DA to cleave the acetate groups on the moiety, releasing an intermediate H_2_DCF which reacts with ROS in its vicinity to form a fluorescent product, 2′,7′-dichlorofluorescein (DCF) [44]. A549 and A375 cells were seeded in 12-well plates (2.5 × 10^3^ cells/well) and allowed to adhere for 24 h. After adhesion, we treated cells as described in experimental protocol. In the next incubation period, cells were collected, washed twice with PBS, and incubated in PBS containing H_2_DCF-DA (10 μM). Cell fluorescence was evaluated after 20 min at 37 °C, using a fluorescence-activated cell sorter (FACSscan; Becton Dickinson, Franklin Lakes, NJ, USA), and was analyzed by Cell Quest software (Becton Dickinson, Milan, Italy).

Mitochondrial ROS levels were evaluated by the uptake of MitoSox-Red. Both cell lines were seeded in 12-well plates with 2 × 10^5^ cells/well. After treatment, cells were stained with MitoSox-Red (2.5 μM) for 15 min in the dark at 37 °C. Later, after collecting and washing with PBS, cells were gently re-suspended in PBS and red fluorescence was detected by flow cytofluorometry (FACScan; BD Biosciences, San Jose, CA, USA) and analyzed by Cell Quest software. MitoSOX is a fluorogenic dye for highly selective detection of superoxide in mitochondria of live cells and, once targeted to mitochondria, it is oxidized only by superoxide, exhibiting red fluorescence.

### 4.7. Flow Cytometry Analysis

Caspase 4 presence was detected by fluorescence-activated cell sorting (FACSscan; Becton–Dickinson). A549 and A375 cells were cultured in a 12-well plate (2.5 × 10^3^ cells/well) and, after 24 h of adhesion, they were treated as described above. At the end of the treatments, cells were collected and treated with a fixing solution (4% formaldehyde, 2% FBS and PBS in the presence of sodium azide 0.1%), for 20 min, and then permeabilized with a buffer containing 4% formaldehyde, 2% FBS, Triton X-0.1%, and PBS in the presence of 0.1% sodium azide for 30 min. Then, anti-caspase 4 was added, and anti-goat Texas-Red was used as a secondary antibody. Lastly, cells were washed with a fixing buffer and then detected by flow cytofluorometry and analyzed by Cell Quest software. Results were shown as a percentage of positive cells.

### 4.8. Determination of Hypodiploid DNA

Briefly, cells were seeded (2.5 × 10^3^ cells/well) in a 12-well plate and allowed to grow for 24 h and treated as previously described. To determine the relative DNA content, fixed cells were washed twice with PBS and resuspended in 500 μL of 0.1% sodium citrate buffer, 0.1% Triton X-100, and 50 μg/mL of propidium iodide (PI), incubating the cells for 30 min at 4 °C in the dark. The PI-stained cells were analyzed by flow cytofluorometry, using Cell Quest software. Cellular debris were excluded from the analysis by raising the forward scatter threshold, and the DNA content of the nuclei was registered on a logarithmic scale. Data are expressed as the percentage of hypodiploid region.

### 4.9. Statistical Analysis

Data evaluations and statistical analysis were made with commercially available software GraphPad Prism8 (GraphPad Software Inc., San Diego CA, USA). All results are represented as mean ± S.E.M. of at least three different experiments performed in technical triplicate. Statistical results between the experimental points were obtained thanks to the non-parametric Mann–Whitney U test. The differences were considered significant if *p* values were from <0.01 to 0.05.

## Data Availability

The authors confirm that the data supporting the findings of this study are available within the article.

## References

[1] Mroczko B., Groblewska M., Litman-Zawadzka A. (2019). The Role of Protein Misfolding and Tau Oligomers (TauOs) in Alzheimer’s Disease (AD). Int. J. Mol. Sci..

[2] Oakes S.A., Papa F.R. (2015). The role of endoplasmic reticulum stress in human pathology. Annu. Rev. Pathol..

[3] Moreno-Gonzalez I., Soto C. (2011). Misfolded protein aggregates: Mechanisms, structures and potential for disease transmission. Semin. Cell. Dev. Biol..

[4] Amodio G., Margarucci L., Moltedo O., Casapullo A., Remondelli P. (2017). Identification of Cysteine Ubiquitylation Sites on the Sec23A Protein of the COPII Complex Required for Vesicle Formation from the ER. Open Biochem. J..

[5] Wang M., Kaufman R.J. (2016). Protein misfolding in the endoplasmic reticulum as a conduit to human disease. Nature.

[6] Chen X., Cubillos-Ruiz J.R. (2021). Endoplasmic reticulum stress signals in the tumour and its microenvironment. Nat. Rev. Cancer.

[7] Lin T., Lee J.E., Kang J.W., Shin H.Y., Lee J.B., Jin D.I. (2019). Endoplasmic Reticulum (ER) Stress and Unfolded Protein Response (UPR) in Mammalian Oocyte Maturation and Preimplantation Embryo Development. Int. J. Mol. Sci..

[8] Hetz C., Zhang K., Kaufman R.J. (2020). Mechanisms, regulation and functions of the unfolded protein response. Nat. Rev. Mol. Cell. Biol..

[9] Kaneko M., Imaizumi K., Saito A., Kanemoto S., Asada R., Matsuhisa K., Ohtake Y. (2017). ER Stress and Disease: Toward Prevention and Treatment. Biol. Pharm. Bull..

[10] Cao S.S., Kaufman R.J. (2014). Endoplasmic reticulum stress and oxidative stress in cell fate decision and human disease. Antioxid. Redox Signal..

[11] Coleman O.I., Haller D. (2019). ER Stress and the UPR in Shaping Intestinal Tissue Homeostasis and Immunity. Front. Immunol..

[12] Wang S., Kaufman R.J. (2012). The impact of the unfolded protein response on human disease. J. Cell. Biol..

[13] Abraham T., Pin C.L., Watson A.J. (2012). Embryo collection induces transient activation of XBP1 arm of the ER stress response while embryo vitrification does not. Mol. Hum. Reprod..

[14] Benhamron S., Hadar R., Iwawaky T., So J.S., Lee A.H., Tirosh B. (2014). Regulated IRE1-dependent decay participates in curtailing immunoglobulin secretion from plasma cells. Eur. J. Immunol..

[15] Dandekar A., Mendez R., Zhang K. (2015). Cross talk between ER stress, oxidative stress, and inflammation in health and disease. Methods Mol. Biol..

[16] Hetz C. (2012). The unfolded protein response: Controlling cell fate decisions under ER stress and beyond. Nat. Rev. Mol. Cell. Biol..

[17] Zhang K., Kaufman R.J. (2008). From endoplasmic-reticulum stress to the inflammatory response. Nature.

[18] Bardin E., Pastor A., Semeraro M., Golec A., Hayes K., Chevalier B., Berhal F., Prestat G., Hinzpeter A., Gravier-Pelletier C. (2021). Modulators of CFTR. Updates on clinical development and future directions. Eur. J. Med. Chem..

[19] Amico G., Brandas C., Moran O., Baroni D. (2019). Unravelling the Regions of Mutant F508del-CFTR More Susceptible to the Action of Four Cystic Fibrosis Correctors. Int. J. Mol. Sci..

[20] Fiedorczuk K., Chen J. (2022). Mechanism of CFTR correction by type I folding correctors. Cell.

[21] Pecoraro M., Franceschelli S., Pascale M. (2021). Lumacaftor and Matrine: Possible Therapeutic Combination to Counteract the Inflammatory Process in Cystic Fibrosis. Biomolecules.

[22] Lee D.Y., Lee K.S., Lee H.J., Kim D.H., Noh Y.H., Yu K., Jung H.Y., Lee S.H., Lee J.Y., Youn Y.C. (2010). Activation of PERK signaling attenuates Abeta-mediated ER stress. PLoS ONE.

[23] Abdullahi A., Stanojcic M., Parousis A., Patsouris D., Jeschke M.G. (2017). Modeling Acute ER Stress in Vivo and in Vitro. Shock.

[24] Rozpedek W., Pytel D., Mucha B., Leszczynska H., Diehl J.A., Majsterek I. (2016). The Role of the PERK/eIF2α/ATF4/CHOP Signaling Pathway in Tumor Progression during Endoplasmic Reticulum Stress. Curr. Mol. Med..

[25] Cao S.S., Kaufman R.J. (2012). Unfolded protein response. Curr. Biol..

[26] Hillary R.F., FitzGerald U. (2018). A lifetime of stress: ATF6 in development and homeostasis. J. Biomed. Sci..

[27] Nakanishi K., Sudo T., Morishima N. (2005). Endoplasmic reticulum stress signaling transmitted by ATF6 mediates apoptosis during muscle development. J. Cell Biol..

[28] Eletto D., Boyle S., Argon Y. (2016). PDIA6 regulates insulin secretion by selectively inhibiting the RIDD activity of IRE1. FASEB J..

[29] Amodio G., Moltedo O., Fasano D., Zerillo L., Oliveti M., Di Pietro P., Faraonio R., Barone P., Pellecchia M.T., De Rosa A. (2019). PERK-Mediated Unfolded Protein Response Activation and Oxidative Stress in PARK20 Fibroblasts. Front. Neurosci..

[30] Nishitoh H. (2012). CHOP is a multifunctional transcription factor in the ER stress response. J. Biochem..

[31] Fan Y., Simmen T. (2019). Mechanistic Connections between Endoplasmic Reticulum (ER) Redox Control and Mitochondrial Metabolism. Cells.

[32] Krebs J., Agellon L.B., Michalak M. (2015). Ca(^2+^) homeostasis and endoplasmic reticulum (ER) stress: An integrated view of calcium signaling. Biochem. Biophys. Res. Commun..

[33] Zhang I.X., Raghavan M., Satin L.S. (2020). The Endoplasmic Reticulum and Calcium Homeostasis in Pancreatic Beta Cells. Endocrinology.

[34] An M.Y., Lee S.R., Hwang H.J., Yoon J.G., Lee H.J., Cho J.A. (2021). Antioxidant and Anti-Inflammatory Effects of Korean Black Ginseng Extract through ER Stress Pathway. Antioxidants.

[35] Valdivieso Á.G., Dugour A.V., Sotomayor V., Clauzure M., Figueroa J.M., Santa-Coloma T.A. (2018). N-acetyl cysteine reverts the proinflammatory state induced by cigarette smoke extract in lung Calu-3 cells. Redox Biol..

[36] Ghemrawi R., Khair M. (2020). Endoplasmic Reticulum Stress and Unfolded Protein Response in Neurodegenerative Diseases. Int. J. Mol. Sci..

[37] Di Conza G., Ho P.C. (2020). ER Stress Responses: An Emerging Modulator for Innate Immunity. Cells.

[38] Berkers G., van der Meer R., Heijerman H., Beekman J.M., Boj S.F., Vries R.G.J., van Mourik P., Doyle J.R., Audhya P., Yuan Z.J. (2021). Lumacaftor/ivacaftor in people with cystic fibrosis with an A455E-CFTR mutation. J. Cyst. Fibros..

[39] Wang F., Liu D.Z., Xu H., Li Y., Wang W., Liu B.L., Zhang L.Y. (2014). Thapsigargin induces apoptosis by impairing cytoskeleton dynamics in human lung adenocarcinoma cells. Sci. World J..

[40] Lin Y., Jiang M., Chen W., Zhao T., Wei Y. (2019). Cancer and ER stress: Mutual crosstalk between autophagy, oxidative stress and inflammatory response. Biomed. Pharmacother..

[41] Franceschelli S., Bruno A.P., Festa M., Falco A., Gionti E., d’Avenia M., De Marco M., Basile A., Iorio V., Marzullo L. (2018). BAG3 Protein Is Involved in Endothelial Cell Response to Phenethyl Isothiocyanate. Oxid. Med. Cell. Longev..

[42] Carrizzo A., Moltedo O., Damato A., Martinello K., Di Pietro P., Oliveti M., Acernese F., Giugliano G., Izzo R., Sommella E. (2020). New Nutraceutical Combination Reduces Blood Pressure and Improves Exercise Capacity in Hypertensive Patients via a Nitric Oxide-Dependent Mechanism. J. Am. Heart Assoc..

[43] Pecoraro M., Marzocco S., Franceschelli S., Popolo A. (2022). Trastuzumab and Doxorubicin Sequential Administration Increases Oxidative Stress and Phosphorylation of Connexin 43 on Ser368. Int. J. Mol. Sci..

[44] James J., Fiji N., Roy D., Andrew M.G.D., Shihabudeen M.S., Chattopadhyay D., Thirumurugan K. (2015). A rapid method to assess reactive oxygen species in yeast using H2DCF-DA. Anal. Methods.

